# Underlying mechanisms of change in cancer prevalence in older U.S. adults: contributions of incidence, survival, and ascertainment at early stages

**DOI:** 10.1007/s10552-022-01595-6

**Published:** 2022-07-07

**Authors:** I. Akushevich, A. Yashkin, M. Kovtun, A. I. Yashin, J. Kravchenko

**Affiliations:** 1 Center for Population Health and Aging, Duke University, Durham, NC USA; 2 Department of Surgery, Duke University School of Medicine, Duke University, Durham, NC USA

**Keywords:** Time trends, Decomposition, Partitioning, Incidence, Survival, Stage ascertainment

## Abstract

**Purpose:**

To quantitatively evaluate contributions of trends in incidence, relative survival, and stage at diagnosis to the dynamics in the prevalence of major cancers (lung, prostate, colon, breast, urinary bladder, ovaries, stomach, pancreas, esophagus, kidney, liver, and skin melanoma) among older U.S. adults age 65 +.

**Methods:**

Trend partitioning was applied to the Surveillance, Epidemiology, and End Results Program data for 1973–2016.

**Results:**

Growth of cancer prevalence in older adults decelerated or even decreased over time for all studied cancers due to decreasing incidence and improving survival for most of cancers, with a smaller contribution of the stage at cancer diagnosis. Changes in the prevalence of cancers of the lung, colon, stomach, and breast were predominantly due to decreasing incidence, increasing survival and more frequent diagnoses at earlier stages. Changes in prevalence of some other cancers demonstrated adverse trends such as decreasing survival in localized and regional stages (urinary bladder and ovarian) and growing impact of late-stage diagnoses (esophageal cancer).

**Conclusion:**

While decelerating or decreasing prevalence of many cancers were due to a beneficial combination of decreasing incidence and increasing survival, there are cancers for which decelerating prevalence is due to lack of improvement in their stage-specific survival and/or increasing frequency of diagnosis at advanced stages. Overall, if the observed trends persist, it is likely that the burden associated with cancer prevalence in older U.S. adults will be lower  comparing to projections based on constant increasing prevalence have previously estimated.

**Supplementary Information:**

The online version contains supplementary material available at 10.1007/s10552-022-01595-6.

## Introduction

More than half of all cancer survivors in the United States are older than 65 years and the number of older survivors is rising [1] thus posing a challenge to the delivery of quality cancer care [2]. Changes in cancer prevalence result from the interplay of the dynamics of cancer incidence, survival, and stage ascertainment; therefore, evaluation of the mechanisms of changes in prevalence over time provides valuable information on the effectiveness of cancer prevention, early diagnosis, and treatment. Furthermore, prevalence is a measure of disease burden in the population that allows for the planning of health care needs [3]. Information on cancer burden in the older U.S. population can be used in analyses of health expenditures, morbidity, quality of life, and life expectancy. It is increasingly recognized that older survivors have complex healthcare needs, but these are poorly understood [4]. At present, little is known about the burden of cancer and comorbidity among the older adults (i.e., especially those aged 85 +) and about the dynamics of this burden over time [5].

While studies on prevalence are common for chronic diseases characterized by long duration (e.g., diabetes [6], Alzheimer’s disease [7], or osteoarthritis [8]), studies on cancer prevalence are less common with more emphasis placed on cancer incidence or mortality, especially in older U.S. adults. This could, partially, be due to the complexity of interpretating prevalence trends which result from three distinct interrelated processes: dynamics in cancer incidence, survival, and the average cancer stage at time of diagnosis. Of these, incidence and survival directly determine the final prevalence trend while stage at time of diagnosis acts indirectly. For example, an increase in cancer incidence (negative health effect) as well as an increase in survival (positive health effect), would drive prevalence up; however, the policy implications between the two events would differ. Similarly, improved early-stage ascertainment (e.g., resulting from more and/or better screening) would lead to increased prevalence through increased incidence (e.g., the disease is identified earlier) and increased survival (e.g., disease is identified both earlier and at an easier to treat stage). However, the health policy takeaway is a positive one, contrary to what increasing prevalence may suggest.

The partitioning analysis [9–12] used in this study, generates models of cancer prevalence, incidence, survival and stage ascertainment and then provides quantitative estimates of the relative fractions of the contributions of the above components to the total change in cancer prevalence. Together, changes in the relative magnitudes of these components provide a quantitative picture describing the epidemiologic causes of prevailing prevalence trends and how these have changed over time in response to changes in screening, treatment and exposure to risk-related factors (e.g., smoking, asbestos exposure, etc.). This allows researchers to identify both positive trajectories in the epidemiology of a specific cancer (e.g., falling incidence, increasing survival, higher proportion of cases diagnosed at lower stages) as well as potential areas of concern requiring urgent attention from the medical and health policy communities. In a time when improved cancer survivorship is becoming a prominent challenge to the U.S. healthcare system, such estimates can provide information invaluable in preparing for the expected increase in cancer survivors [13, 14]. Our study focuses on the U.S. population of older adults (aged 65 +) because this population has the highest cancer prevalence among all age groups and, due to the high rates of chronic morbidities in this age group, represents the highest potential challenge for maintaining the health and wellbeing of cancer survivors. Furthermore, the overwhelming majority of this population is eligible for health insurance provided by the Medicare social insurance system. This reduces the bias associated with differences in access to and affordability of cancer care.

## Data and methods

Data were drawn from the Surveillance, Epidemiology, and End Results program (SEER), 1973–2016 [15]. SEER information on the date and age of diagnosis, cancer type, stage at diagnosis, and the number of survival months with or without death at the end of follow-up. All diagnosed cases of cancers of interest available in SEER data were used. The cases diagnosed in 1992 + were used to calculate all three measures (incidence, survival, and stage-specific fractions), and cases before 1992 contributed to survival starting from 1992 and provided reasonable estimates of cancer prevalence in 1992–2016. The following codes of SEER site records ICD-O-3 were used for analysis: cancer of lung and bronchus (C340–C349), breast (C500–509), prostate (C619), colon (C180, C182–C189, C199), pancreas (C250–C259), urinary bladder (C670–C679), esophagus (C150–C159), liver (C220), kidney (C649, C659), stomach (C160–C169), ovarian (C569), and skin melanoma (C440–C449). We included the top ten of most frequent cancers (Fig. 6.2 of Ref. [16].), bladder cancer for which the partitioning methods were applied earlier [12], and melanoma as potentially curable and preventable cancer with rapidly increasing time trend [17]. SEER*Stat software [18] was used to evaluate age-adjusted prevalence that was used for comparisons with prevalence proportions calculated using the partitioning approach.

### Partitioning analysis

The partitioning approach [9–12] is based on an explicit representation of disease prevalence with no simplifying assumptions. The full mathematical derivation of the partitioning approach and its application to lung cancer is available in the study by Akushevich et al. [11]. In brief, the method predicts cancer prevalence and decomposes (or partitions) their time trends into their constituent components by calculating the relative impact each component has on the overall trend as well as inter-temporal changes in the strength and direction of these impacts. Specific outcome measures are age-specific and age-adjusted prevalence; the constituent components are incidence, relative survival, and frequency of cancer stage at diagnoses.

The model of cancer prevalence is based on the idea that the probability of being prevalent at a given age requires being incident before and survival after that age. Time trends in age-adjusted prevalence are defined as their derivatives with respect to time $y$. Explicit calculation of time trends in age-adjusted prevalence results in $P^{\prime}(y)/P(y) = T_{inc} (y) + T_{\pi } (y) + T_{S} (y)$, representing changes in incidence, cancer stage at the time of diagnosis, and relative survival, respectively. Three stages (localized, regional, and distant) are defined by SEER or modeled from AJCC stages (for prostate cancer). Explicit expressions for partitioning components of cancer age-adjusted prevalence are given in Eqs. 5 in the study by Akushevich et al. [11]. Each component is to be interpreted as the rate of change at any point in time (increasing if > 0 and decreasing if < 0) with the magnitude of the effect indicating the speed of the change. The sum of the contributions adds to + 100% if the decomposed prevalence is increasing and to − 100% if the decomposed prevalence is decreasing at a given point in time.

### Model estimation

The quantities of interest [i.e., $T_{{{\text{inc}}}} (y)$, $T_{\pi } (y)$, and $T_{S} (y)$] are expressed in terms of the derivatives of the respective functions with respect to time. In our approach, we used explicit analytic parameterizations for all functions for which derivatives are needed. Therefore, we calculated the derivatives analytically. This allowed us to avoid dealing with possible numeric instabilities occurring when derivatives are evaluated numerically. The analysis involved the design and estimation of three models: (i) for the incidence rate, (ii) for frequencies of stage at diagnosis, and (iii) for the probability of relative survival after cancer diagnosis.

The incidence rate providing the distribution of age at cancer onset was modeled using the Armitage-Doll model with additional individual predisposition modeled by gamma or inverse Gaussian distributions (for incidence) [19]. This approach results in explicit parameterization of cancer incidence using four parameters, $I(x) = \frac{{x^{m - 1} }}{{c^{m} \left( {1 + n\sigma^{2} c^{ - m} m^{ - 1} x^{m} } \right)^{1/n} }}$, where four model parameters have the following meaning: $m$ is the number of carcinogenesis stages; $c$ (in years) is the scale parameter; $\sigma^{2}$ is the variance of the random predisposition to cancer risk; and $n$ is the parameter describing the shape of frailty distribution (e.g., $n = 1$ for gamma-distribution or $n = 2$ for inverse Gaussian distribution).

Frequencies of stages at cancer diagnosis are parameterized as quadratic functions of age. Three stages are distinguished in SEER data for a cancer: local $\left( {s = 1} \right)$, regional $\left( {s = 2} \right)$, and distant $\left( {s = 3} \right)$. Thus, the frequencies of being diagnosed at stage $s = 1$ and $s = 2$ are modeled as $f_{s} = f_{s0} + f_{s1} \tau + f_{s2} \tau^{2}$ and $f_{3} = 1 - f_{1} - f_{2}$.

The Weibull model for time after disease onset is used for relative survival, $S_{rel} = \exp \left( { - \alpha x_{\tau }^{\gamma } } \right)$, where $x_{\tau }$ is the survival time. Model parameters are expressed in terms of $\sigma$ and $\mu$ ($\alpha = \exp ( - \mu /\sigma )$ and $\gamma = 1/\sigma$) which are parameterized as quadratic functions of age at diagnosis $\mu = \mu_{0} + \mu_{1} \tau + \mu_{2} \tau^{2}$ and $\sigma = \sigma_{0} + \sigma_{1} \tau + \sigma_{2} \tau^{2}$. This approach was used for all cancer sites except prostate and breast, and skin melanoma for which we use the model in terms of $\alpha$ and $\sigma$. The latter model allows for relative survival for certain cancer sites exceeding 100% (or > 1.00): i.e., when survival of patients with certain early-stage cancers was better than survival of individuals in general U.S. population aged 65 +, that is observed for these cancer sites.

For any model we used B-spline functions to model relationships between year-specific model parameters and evaluate *y*-dependence of the function. We use B-splines of 3rd degree defined by equidistant set of knots, 4 inner and two outer knots are used. Any model parameter is represented

$$p(y) = b_{0} B_{0} (y) + b_{1} B_{1} (y) + b_{2} B_{2} (y) + b_{3} B_{3} (y) + b_{4} B_{4} (y) + b_{5} B_{5} (y)$$

for the estimation region $1992 \le y \le 2016$. The peaks of B-splines $B_{1 - 4} (y)$ are located at years 1992, 2010, 2008, and 2016, respectively. We use the restriction $b_{0} = b_{1}$ and $b_{4} = b_{5}$ which is justified by the fact that $B_{0} (y)$ and $B_{5} (y)$ are nonzero only in a small part of the estimation region. B-splines allow explicit calculation of derivatives without requiring additional simplifying assumptions.

All three models (incidence, stage frequency, and relative survival) were estimated separately and obtained fits were used to construct the model for cancer prevalence for each cancer site. The least-square approach was used to estimate model parameters for incidence and stage-specific fractions at diagnoses and the likelihood-based approach [20] developed specifically for estimating models for relative survival is used in our study. To assess the goodness-of-fit of our model, age-patterns of cancer incidence (averaged over 1992–2016) and time patterns of age-adjusted measures for incidence, stage frequencies, and relative survival are shown in the 12 cancer-specific panels of Supplementary Fig. 1. The predicted prevalence model calculated based on partitioning approach was compared to the estimates provided by SEER (available through SEER*Stat software). The SEER*Stat estimates for cancer prevalence were generated using two alternative lookback periods: 18 years, the longest period available for all years of cancer initial diagnosis; and up to 23 years, the longest period available in the data, although not for all years of cancer initial diagnosis.

## Results

**Fig. 1:**
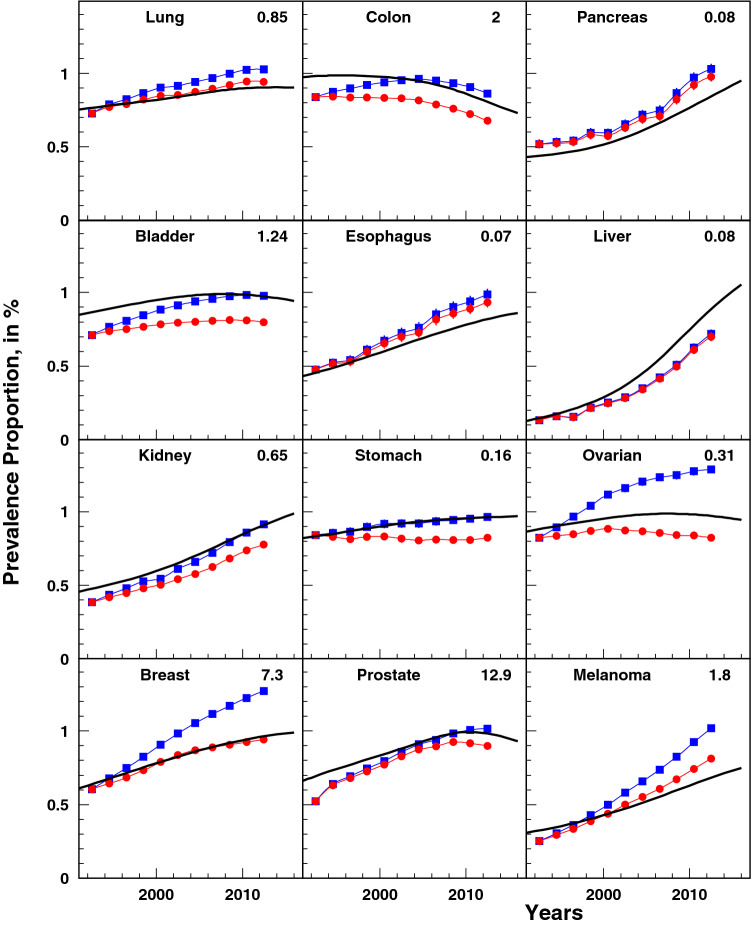
Estimates of cancer prevalence. Age-adjusted prevalence using our model (solid black line), age-adjusted prevalence using SEER*Stat using an 18-years look-back period (red line with filled circles), and age-adjusted prevalence using SEER*Stat using an up to 23-years look-back period (blue line with filled squares). The value on each plot is a rescaling factor. Actual prevalence is obtained by multiplication of this factor and the value taken from *Y*-axis of the plot. (Color figure online)

The trends of cancer-specific prevalence were calculated using the partitioning approach and compared to corresponding estimates provided by the SEER*Stat tool (Fig. 1). Adding the look-back years in SEER*Stat estimates increases the prevalence by identifying additional patients who were still surviving persons after being diagnosed with a cancer over the look-back period. Our model, which calculates cancer prevalence outside the bounds of the look-back periods, yielded the highest prevalence levels. The agreement between empirical data and our model was good for all cancers: the shape of the model reflected respective empirical patterns for 18 years, with differences in magnitude reflecting the effect of accumulated prevalence from earlier years as expected [12].

**Fig. 2:**
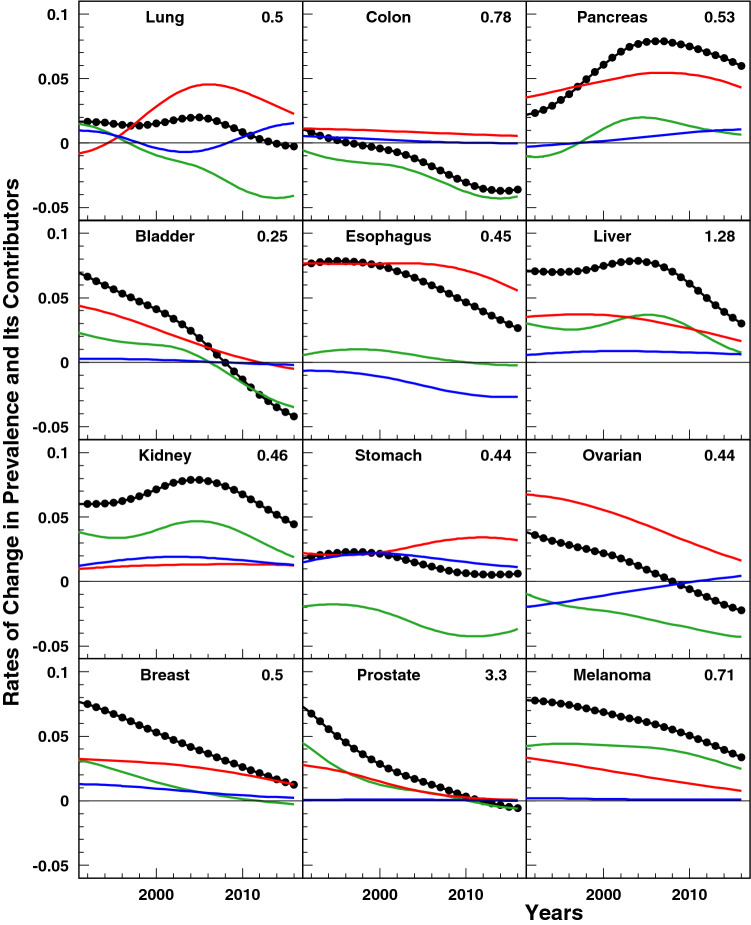
Partitioning of age-adjusted prevalence of studied cancers. Color curves represent the contributions of incidence (green), survival (red), and frequency at diagnosis (blue). (Color figure online)

Cancer prevalence estimated from our model monotonically increased for all cancer sites with exceptions for four cancers for which a maximum in prevalence was reached with subsequent decline. These were: cancers of colon (with maximum in 1999), urinary bladder (in 2008), ovarian (in 2008), and prostate (in 2010) (Fig. 1). Cancers of the pancreas, esophagus, liver, kidney, and melanoma had the most pronounced increases in prevalence over time. However, the relative rates of change in cancer prevalence (Fig. 2; black dots) show that these prevalence increases have been slowing down. Even for pancreas and kidney cancers which had periods of increases in their rates of change in 1992–2013 and 1992–2008, respectively, recent trends show a deceleration in the growth of the prevalence proportion.

Each of the three prevalence decomposition components, incidence, relative survival, and frequencies of stage at diagnosis is also shown in Fig. 2. Together, all three components add up to the curve representing relative rates of change in cancer prevalence. Interpretation can be illustrated, for example, for cancer of the esophagus. Its prevalence increased over the entire study period (black dots all above 0.00) but at a decreasing rate (negative slope on black dot series). The primary contributor was survival (red line) which has been steadily improving (red line above 0.00) over the entire study period. Growing incidence (green line) played a relatively small role in the prevalence increase between 1992 and 2008 (green line above 0.00) after which incidence started decreasing (green line below 0.00) acting to delay the rate of esophagus cancer prevalence growth. The strong effect of survival improvement (red line) is hampered by a strong and negative trend in early-stage diagnosis (blue line below 0.00) demonstrating the relationship between a higher proportion of esophageal cancer cases being diagnosed at later stages and survival. Such a pattern suggests need for improved screening efforts as the only negative aspect of the observed prevalence increase is a higher proportion of individuals being diagnosed at later stages.

Supportive information on explicit trends of partitioning components obtained empirically and in the model is shown in Supplementary Fig. 1. Empirical trends showed that age-adjusted (65 +) incidence rates for cancers of the lung, colon, bladder, stomach, and ovarian declined over the 1992–2016 period. Only skin melanoma had increasing incidence rates during entire study period. Other cancers had periods of both increasing and decreasing incidence rates (Supplemental Fig. 1). The results of partitioning analysis (Fig. 2) show that changes in incidence rates were the main contributors to the deceleration of the growth in the prevalence of many cancers. Most substantially incidence contributed to the deceleration in the growth of prevalence proportions for cancers of the lung, colon, urinary bladder (since 2005), stomach, ovaries, and breast (since 2010) as well as the decline in the prevalence of cancers of the colon, bladder (since 2005), ovarian (since 2008), and prostate (since 2011). Although trends in incidence differ, the rate of change in the incidence effect goes down for most of the studied cancers (Fig. 2), although for cancers of kidney, liver, and pancreas the decline starts on or about 2005.

Empirical trends of 1- and 3-year survival showed gradual improvements in relative survival among older adults age 65 + for all cancers except urinary bladder and ovarian (for local and regional stages), liver (for regional and distant stages), and prostate (local and distant) (Supplemental Table 1). Partitioning analysis showed that the relative survival from all cancers had a beneficial trend and pushed cancer prevalence up (Fig. 2). However, the impacts of better survival on the prevalence trend were slowing down over time for all cancers except kidney cancer. The most rapid decline in the effect of survival was detected for urinary bladder and ovarian cancers.

Empirical trends showed that over time, in older adults age 65 + , cancers of breast, prostate, skin melanoma, colon, urinary bladder, liver, and kidney were more often diagnosed at early (i.e., localized) stages (Supplemental Table 1). However, cancers of lung, pancreas, stomach, ovaries, and esophagus were more often diagnosed at distant rather than localized or regional stages, and this trend was growing for lung, stomach and especially esophageal cancers (Supplemental Table 1). Compared to the contributions of incidence and relative survival, the contribution of stage at ascertainment to the dynamics of cancer prevalence were less pronounced for most studied cancers. This contribution was minimal for cancers of colon, urinary bladder, prostate, and skin melanoma, but the effect of stage at ascertainment on the dynamics of cancer prevalence overcame the impacts of incidence for pancreatic cancer (since 2013) and was comparable to impact of relative survival for kidney cancer (Fig. 2). A disadvantageous and relatively strong in magnitude effect of stage ascertainment was detected for esophageal cancer (i.e., the impacts of cancer being diagnosed at advanced stage) (Fig. 2).

## Discussion

Using a partitioning approach [11, 12], we quantitatively evaluated contributions of cancer incidence, relative survival, and early-stage diagnoses to the changes in cancer prevalence in older U.S. adults aged 65 +. In this study, partitioning of prevalence was analyzed together with empiric patterns of cancer incidence and stage-specific relative survival thus providing more detailed information on the mechanisms of changes in the prevalence of various cancers in the U.S. For all studied cancers, growth of prevalence decelerated over time and for cancers of the colon, urinary bladder, ovaries, and prostate, the prevalence passed its respective maximum resulting in declines over recent years. Trends in cancers of lung, colon, stomach, and breast reflect positive reasons for the observed prevalence change: decreasing incidence and increasing survival together with increases in diagnoses at earlier stages over time. However, prevalence trends of certain cancers reflect adverse patterns. Specifically, cancer of urinary bladder shows decreasing survival in localized and regional stages, ovarian cancer shows decreasing survival in localized and regional stages, although with an increase in early-stage diagnoses, and esophageal cancer shows an increasing impact of late-stage diagnoses.

### Why it is important to understand underlying mechanisms of cancer trends in older U.S. adults

Aging of the U.S. population is a well-recognized factor contributing to cancer burden [16, 21]. As a population group, older adults are characterized by higher cancer prevalence combined with higher levels of functional and cognitive impairment, multiple coexisting morbidities, and a higher risk of treatment side effects that could bias treatment choice towards less aggressive (and potentially  less effective) treatment choices [22]. In certain cases some conservative treatments, such as active surveillance, can potentially be the optimal initial choice of treatment in the 65 + age group; at least in the early stages of some cancers [23]. This is one case where trend partitioning could be of particular use as it allows researchers to model historical and current trends in the stage at which a given cancer is identified on average, make future forecasts based on these models and bring any adverse dynamics (such as the growing levels of late-stage esophageal cancer identified in this study) to the attention of the healthcare community.

Furthermore, older adults continue to be an understudied population group. Cancer patients age 65 + are largely excluded from clinical trials, slowing the spread of insight on the unique needs of older patients, including long-term survivors [2, 24, 25]. This point is emphasized by the Institute of Medicine as an important part of addressing the challenges in the delivery of quality cancer care [24]. Trend partitioning does not require specialized studies and can use the significant body of epidemiological data available. More detailed datasets allow for more components in the decomposition, but even basic trend decompositions such as the one conducted in this study are highly informative. For example, an increase in incidence combined with an increase in proportion of ascertainment at very early stages may indicate improvements in diagnostic technology rather than a potential health crisis. Similarly, an increase in cancer-related mortality could be the result of delayed mortality stemming from improvements in survival in earlier time periods [12]. The long-term success/failures of new screening initiatives and/or treatment regimens can be assessed in a similar manner (as can the effects of differences in practice such as cancer staging). Access to such information would be invaluable in informing the formulation of health policy.

### Dynamics of cancer prevalence among older U.S. adults

The main driving forces underlying the prevalence trends observed in our study were a deceleration in incidence increase and improved relative survival for the majority of cancers studied. If current trends persist, we expect a substantial deceleration in the prevalence of many cancers in the U.S. population of older adults age 65 + within the next decades. For many cancers prevalence growth has slowed down (lung, stomach, and breast) or even started to decline (colon, prostate, ovarian, and urinary bladder). These trends are not in a full agreement with recent projections suggesting that the prevalence of cancer survivors will increase from 15.5 million to 26.1 million (across all age groups) from 2016 to 2040, with the largest growth of cancer survivors in the older population [2]. The same study estimated that compared to 1975, the 2040 projected cancer prevalence will be sixfold higher for those aged 65–74 years, tenfold higher for ages 75–84 years, and 17-fold higher for ages 85 + [2]. However, this study held the rates of cancer incidence and survival constant for the whole period of the projection, while in our approach we incorporated the dynamics of cancer incidence, relative survival, and stage at cancer diagnosis when analyzing changes of prevalence of each cancer.

### Dynamics of cancer incidence and its role in cancer prevalence trends

Deceleration of growth of cancer prevalence obtained in our study can be explained, to a great extent, by the deceleration of the increase or actual decrease of cancer incidence over time such as observed for cancers of lung, colon, stomach, ovaries, urinary bladder, and breast. Effectiveness of ongoing preventive strategies substantially contributed to the deceleration of incidence observed over recent decade. They include, but are not limited to, decreasing smoking prevalence (affects risk of lung, urinary bladder, kidney, liver, colon, and stomach cancers, with the respective percent of smoking-attributable deaths estimated at 80.2%, 44.8%, 16.8%, 23.6%, 9.7%, and 19.6%, respectively [26]), implementation of gastric cancer prevention programs that includes screening for *Helicobacter pylori* infection and its treatment [27], and decreasing prevalence of hepatitis B infection and its increasing vaccination rate (for liver cancer [28]). However, persisting or even increasing prevalence of metabolic syndrome, obesity, and hepatitis C, as well as a poor diet, low physical activity, and other risk factors still slow the deceleration of cancer incidence.

### Dynamics of relative survival and its role in cancer prevalence trends

The trends of relative survival obtained in our study were beneficial for all studied cancers, though improvement in relative survival has slowed down within recent decade for many cancers. The beneficial trends of relative survival could decline over time because of accumulation of cancer patients who benefited from increased survival in earlier years and, therefore, died later [12]. For example, it has been shown that the number of cancer survivals among older U.S. adults grew over time, with gender-specific differences widening at older ages: there was 5–8% more male than female cancer survivors at ages 70–79, 11% more at ages 80–89, and 12% more at age 90 + (with 37% of men aged 90 + being cancer survivors [2]. Slowing improvement or even decline in relative survival detected for several cancers (e.g., bladder, liver, ovarian) in our study can be the result of the accumulation of delayed mortality in individuals diagnosed early in the study time-period and benefiting from improved survival at the time of their diagnosis. Targeted partitioning of cancer mortality [12] would be required to further analyze this hypothesis.

The contributions of slowing improvements in survival to dynamics of prevalence were higher for cancers of prostate, ovaries, and urinary bladder. These effects were also observed for empiric trends of stage-specific survival of these cancers: e.g., decreasing trends of prostate cancer survival in epidemiologic analysis for localized and distant stages (Supplemental Fig. 1). Another study showed decreases in survival (nonstage-specific) among patients with prostate cancer from 2006/2010 to 2011/2016: a 5-year survival decreased for 1.3% for ages 65–79 and for 7.2% for patients aged 80 + [29]. It has been suggested that lower survival at older ages might be due to a more rapid development of resistant prostate cancer, reduced ability to receive cancer therapies, and the impact of comorbidities [29, 30]. However, that cannot explain the changes in prostate cancer survival among older patients during the recent decade, as well as the negative dynamics of survival at localized stages observed in our study.

The trends of ovarian cancer survival among females age 65 + show that while there are positive contributions of the decreasing role of incidence and increasing frequency of earlier stage diagnoses of ovarian cancer, the stage-specific relative survival of patients with localized and regional tumors decreased since 2003. While publications on survival trends among older patients with ovarian cancer remain sparse, a recent study showed that females with ovarian cancer age 75 + had a lower frequency of stage-appropriate surgery (OR 0.58, 95% CI 0.40–0.83) and multi-agent chemotherapy (OR 0.27, 95% CI 0.17–0.41) [31] than younger patients. In general, there has been a decline in the number of women receiving the guideline-concordant recommendation of primary debulking surgery followed by at least 6 cycles of systemic platinum- based chemotherapy or surgery and chemotherapy [32]. Furthermore, the standard treatment modalities were less often applied to older ovarian cancer patients compared to younger patients resulting in poorer survival [33]. In addition, women with more comorbidities may receive surgery alone resulting in a lower survival rates.

We also observed a tendency for decreasing 3-year survival among older patients with localized or regional cancer of urinary bladder. This is in agreement with a study showing that treatment developments over the recent three decades did not result in improvements in mortality from localized or regional cancers of urinary bladder, especially among older patients [34]. The mechanisms of this trend are not well understood. At least partially, it could be explained by more frequent severe comorbidities in older patients resulting in less aggressive cancer treatment and/or higher mortality due to comorbid conditions. Extensive efforts were made to improve the accuracy of both the cross-sectional and molecular imaging modalities of bladder cancer to optimize management and treatment decisions [35], however effects of these efforts have not yet translated to changes in survival. Note, that the new staging system groups regional lymph node metastasis in the true pelvis and/or the common iliac nodes (N1–3) within stage III (rather than being previously grouped within stage IV) [36, 37]; however, these changes cannot explain the observed trends of survival among patients with localized or regional cancer.

One powerful factor that influences stage-specific survival time trends could be an increasing prevalence of poorly differentiated tumors. For ovarian and urinary bladder cancers recent declines of stage-specific survival were detected (Fig. 2 and Supplementary Fig. 1). These two cancers have a high prevalence of undifferentiated grade tumors at diagnoses and their prevalence increases over time [38, 39]. Our estimates using SEER data show that the prevalence of undifferentiated tumors at regional stage in 2000, 2005, 2010, and 2015 were 32%, 46%, 62%, 78%, respectively, for urinary bladder cancer and 9%, 13%, 23%, 32% for ovarian cancer. For other cancers (maybe, excluding kidney cancer) the frequencies of undifferentiated tumors were lower and/or did not change substantially over time. This finding requires further detailed analysis and should be interpreted with caution because of changes to the coding system guidelines that likely affected the way grade was interpreted by registrars and documented in the grade/differentiation variable [38, 40].

### Stage at cancer diagnosis and its role in the dynamics of cancer prevalence

Compared to contributions of relative survival and incidence, changes in stage at cancer diagnoses contributed least to the dynamics of cancer prevalence for most of the cancers in our study except for cancers of the pancreas, kidney, stomach (through increasing frequency of early-stage diagnoses), and esophagus (through increasing frequency of late-stage malignancies). As expected, cancers with well-established screening strategies (e.g., prostate, breast, and skin melanoma), showed minimal changes in the impact of improvements in ascertainment.

Cancers of the pancreas and kidney are relatively rarely diagnosed at early stages and screening for these cancers is not feasible in the general population [41, 42]. Major barriers include relatively low prevalence, the potential for false positives and over-diagnosis, unlikely use of computer tomography for population screening due to cost, radiation dose, and increased potential for other incidental findings, and varying accuracy of ultrasound screening depending on tumor size [43, 44]. Screening for pancreatic cancer is recommended for high-risk groups starting from age 50 (with the use of endoscopic ultrasound and/or magnetic resonance imaging) [41, 45], and screening for kidney cancer (with the use of computer tomography or magnetic resonance imaging) is recommended for individuals with a known heritable syndrome associated with the development of renal cell carcinoma [46, 47]. For pancreatic cancer, imaging-based screening in groups at high familial risk can detect cancer with some evidence of minimal harm. However, while only about 10% of cases have a familial basis, no data are available on the general population and the impact of screening on other high-risk populations (e.g., individuals with diabetes, smoking history, or chronic pancreatitis) remain unknown [44]. At the same time, early detection of smaller tumors allows for the use of minimally invasive techniques (e.g., robotic or laparoscopic partial nephrectomy and tumor ablation) and for reducing rates of open surgery with associated high levels of morbidity [44]. This may be reflected in the increasing levels of early-stage diagnoses for pancreatic and kidney cancer observed in our study.

The potential effectiveness of a stomach cancer prevention program that includes *Helicobacter pylori* screening and treatment is dependent on individual level of cancer risk [27]. While population-based screening for *H. pylori* and eradication of this infection has been shown to be cost effective in countries with high incidence of stomach cancer, in the U.S. this screening strategy is thought to be generally unwarranted [48, 49]. Although stomach cancer screening is recommended in some U.S. regions of high disease prevalence, screening is not routinely performed in the U.S. [50] and no clear guidelines for endoscopic gastric cancer screening are currently established [27]. There is some evidence that population-based *H. pylori* serology screening could be potentially cost-effective for populations with stomach cancer rates as low as 4.2/100,000 [49].

Esophageal cancer demonstrated slowing improvement in survival combined with increasing levels of diagnoses at advanced stages. Both effects substantially contributed to deceleration in cancer prevalence among older U.S. adults. Although recent analysis of SEER data showed that 5-year survival rates for all age patients with esophageal cancer increased from 1973 to 2010 (for both localized and regional stages), information on survival among older patients remains sparse [51]. It has been speculated that survival of patients with esophageal cancer was slowly improving due to better surgical techniques, adjuvant therapy, and increasing use of upper gastrointestinal endoscopy for early diagnosis [51, 52]. However, it seems that this may not be the case for older patients for whom a deceleration in survival could be associated with changing patterns of histotype-specific incidence with esophageal adenocarcinoma rates surpassing the rates of squamous cell carcinoma [51, 53]. These patterns could contribute to the observed dynamics of early-stage diagnoses. In the U.S., the only data to guide the deployment of standard and novel screening and surveillance programs for esophageal cancer are derived from population-based cohort studies from the North-European countries and institutional cohort studies from tertiary care centers [54]; however, these data may not reflect the patterns across the U.S. One opportunity to fill these knowledge gap is by studying long-term outcomes in patients with Barrett’s esophagus (the only known premalignant condition for esophageal carcinoma) from administrative health claims data, as well as evaluating the impacts of risk factors and healthcare utilization, to guide future efforts to develop surveillance programs that reduce morbidity and mortality of esophageal carcinoma in the U.S. [54]. Future studies should also address potential causes of changes in survival among older patients with histotype-specific esophageal cancer.

Our decomposition identified four cancers with strong trends in the contribution of early-stage ascertainment which could be potentially leveraged into future improvements in incidence and survival through targeted policy action. Improved screening for cancers of the kidney, pancreas, and esophagus remains the greatest challenge as the optimal screening modality and screening strategies are yet to be determined [43]. While more frequent use of cross-section imaging can result in these malignancies being diagnosed at earlier stages in the general population [55], individual patient risk-stratification based on a combination of risk factors may improve screening efficiency and minimize harms by identifying high-risk groups, including the development and validation of risk prediction models containing phenotypic and genotypic data, to explore the potential benefits of targeted screening [43].

### Why are the studies on cancer prevalence trends relatively rare compared to the studies on incidence or mortality?

Although many studies focus on cancer mortality trends in the U.S. [56–59], studies on cancer prevalence including analyses of dynamics of epidemiologic characteristics that impact prevalence trends are less common [2, 60]. In part, this is due to a lower availability of data on disease prevalence, while mortality data are widely available (e.g., in the Multiple Cause of Death datafiles). Even when the data on cancer prevalence can be obtained (e.g., from SEER-Medicare data), the results requires complex interpretation compared to the results on incidence or mortality [10] because prevalence is based on both disease incidence and its duration, and a high prevalence of disease in a population could reflect high incidence, or better survival, or both (and a low prevalence could indicate low disease incidence, or worth survival, or both). Partitioning analysis we used provides: (i) high-precision estimates of time trends of cancer prevalence; (ii) explanations of these trends based on the trends of cancer incidence, stage-specific relative survival, and ascertainment of cancer at early stages; and (iii) interpretation of the obtained results and identification of the groups of cancers with similar patterns. This method reveals the mechanisms of interrelations between cancer incidence, prevalence, and relative survival, thus being an important step in assessing current and future cancer burden, identification of potential problem areas that requires more attention, and evaluation of how these important epidemiological characteristics respond to implemented health interventions over time.

### Study limitations

The SEER database used in this study represents approximately 28% of the U.S. population and underrepresents certain racial/ethnic groups and geographic regions. In this study we investigated cancer sites without further detailing by histotypes; however, cancers of different histotypes (e.g., adenocarcinomas and squamous cell carcinomas, subtypes of ovarian or breast cancers, etc.) are distinct by their risk factors, biology, time trends, populations at risk, treatment, and survival; therefore, their contributions to prevalence trends also differ. Further, our calculations were based on empiric parametric models for partitioning components, and although the models were constructed based on accepted models some statistical uncertainties still remain. In addition, the model for cancer survival in the most recent years can be less precise because of a limited survival time available. SEER data do not provide individual-level variables on cancer risk factors (e.g., smoking); therefore, the dynamics of these factors were not integrated into partitioning analysis. Finally, we did not perform gender- and race/ethnicity-specific partitioning analysis in this study to keep it focused on various cancer sites; further studies will include more detailed analyses for these populations subgroups.

## Summary

Our analysis shows that the slowing in the increases in the prevalence of many cancers among older U.S. adults over recent decades can be explained by decreasing trends of incidence and improving survival for most cancers, with smaller contributions of the stage at cancer diagnosis. If these dynamics persist, it is likely that cancer burden in older U.S. population of older adults will be lower than projected previously. While decelerating or decreasing prevalence of many cancers are due to a beneficial combination of decreasing incidence and increasing survival, there are cancers for which decelerating prevalence is due to the lack of improvement in their stage-specific survival and/or increasing frequency of diagnosis at advanced stages. Overall, if the observed trends persist, it is likely that the burden associated with cancer prevalence in older U.S. adults will be lower than projections based on constant increasing prevalence have previously estimated; although due to the overall growth of the size of the at-risk population, this burden will still be significant. Although the literature on cancer burden and its trends in older populations remains sparse, clinicians are increasingly recognizing the benefits of a geriatric assessment framework in oncology [5, 61], with the complex health needs of older patients and finding efficient ways to meet the medical surveillance needs of older survivors will become increasingly important [2, 62]. Although a number of interventions have been developed to help survivors cope with cancer, few have targeted older adults and this remains an area of critical need in survivorship science [2, 62].

## Supplementary Information

Below is the link to the electronic supplementary material.

Supplementary File 1 (PDF 221 kb)

## Data Availability

The datasets generated during and/or analyzed during the current study are available in the National Cancer Institute repository, https://seer.cancer.gov/data/.

## References

[1] De Moor JS (2013). Cancer survivors in the United States: prevalence across the survivorship trajectory and implications for care. Cancer Epidemiol Prev Biomark.

[2] Bluethmann SM, Mariotto AB, Rowland JH (2016). Anticipating the “silver tsunami”: prevalence trajectories and comorbidity burden among older cancer survivors in the United States. Cancer Epidemiol Prev Biomark.

[3] Ward MM (2013). Estimating disease prevalence and incidence using administrative data: some assembly required. J Rheumatol.

[4] Bellizzi KM (2012). Double jeopardy? Age, race, and HRQOL in older adults with cancer. J Cancer Epidemiol.

[5] Hurria A (2015). Improving the evidence base for treating older adults with cancer: American Society of Clinical Oncology statement. J Clin Oncol.

[6] Andes LJ (2019). Diabetes prevalence and incidence among medicare beneficiaries—United States, 2001–2015. Morb Mortal Wkly Rep.

[7] Association AS (2019). Alzheimer's disease facts and figures. Alzheimer's Dementia.

[8] O'Neill TW, McCabe PS, McBeth J (2018). Update on the epidemiology, risk factors and disease outcomes of osteoarthritis. Best Pract Res Clin Rheumatol.

[9] Akushevich I (2017). Theory of partitioning of disease prevalence and mortality in observational data. Theor Popul Biol.

[10] Akushevich I (2018). Identifying the causes of the changes in the prevalence patterns of diabetes in older US adults: a new trend partitioning approach. J Diabetes Complic.

[11] Akushevich I (2019). Partitioning of time trends in prevalence and mortality of lung cancer. Stat Med.

[12] Akushevich I (2020). Partitioning of time trends in prevalence and mortality of bladder cancer in the United States. Ann Epidemiol.

[13] Yabroff KR (2019). Minimizing the burden of cancer in the United States: goals for a high-performing health care system. CA Cancer J Clin.

[14] Ellis L (2018). Trends in cancer survival by health insurance status in California from 1997 to 2014. JAMA Oncol.

[15] Whitford GM (1999). Fluoride metabolism and excretion in children. J Public Health Dent.

[16] Manton KG, Akushevich I, Kravchenko J (2009). Cancer mortality and morbidity patterns in the US population: an interdisciplinary approach.

[17] Aggarwal P, Knabel P, Fleischer AB (2021). United States burden of melanoma and non-melanoma skin cancer from 1990 to 2019. J Am Acad Dermatol.

[18] Whitford G (1994). Effects of plasma fluoride and dietary calcium concentrations on GI absorption and secretion of fluoride in the rat. Calcif Tissue Int.

[19] Kravchenko J (2012). Evaluating the number of stages in development of squamous cell and adenocarcinomas across cancer sites using human population-based cancer modeling. PLoS ONE.

[20] Dickman PW (2004). Regression models for relative survival. Stat Med.

[21] Cho H (2013). Assessing non-cancer-related health status of US cancer patients: other-cause survival and comorbidity prevalence. Am J Epidemiol.

[22] Foster JA (2010). How does older age influence oncologists' cancer management?. Oncologist.

[23] Akushevich I (2020). A medicare-based comparative mortality analysis of active surveillance in older women with DCIS. NPJ Breast Cancer.

[24] Levit LA (2013). Delivering high-quality cancer care: charting a new course for a system in crisis.

[25] Unger JM (2006). Impact of the year 2000 Medicare policy change on older patient enrollment to cancer clinical trials. J Clin Oncol.

[26] Siegel RL (2015). Deaths due to cigarette smoking for 12 smoking-related cancers in the United States. JAMA Intern Med.

[27] Thrift AP, El-Serag HB (2020). Burden of gastric cancer. Clin Gastroenterol Hepatol.

[28] Kruszon-Moran D (2020). Prevalence and trends in hepatitis B virus infection in the United States, 2015–2018. NCHS Data Brief.

[29] Siegel DA (2020). Prostate cancer incidence and survival, by stage and race/ethnicity—United States, 2001–2017. Morb Mortal Wkly Rep.

[30] Bernard B (2020). Impact of age at diagnosis of de novo metastatic prostate cancer on survival. Cancer.

[31] Warren JL (2017). Trends in the receipt of guideline care and survival for women with ovarian cancer: a population-based study. Gynecol Oncol.

[32] Thrall MM (2011). Trends in treatment of advanced epithelial ovarian cancer in the Medicare population. Gynecol Oncol.

[33] Schuurman M (2018). Treatment and outcome of elderly patients with advanced stage ovarian cancer: a nationwide analysis. Gynecol Oncol.

[34] Abdollah F (2013). Incidence, survival and mortality rates of stage-specific bladder cancer in United States: a trend analysis. Cancer Epidemiol.

[35] Mirmomen SM (2019). Preoperative imaging for locoregional staging of bladder cancer. Abdom Radiol.

[36] Flaig TW (2018). NCCN guidelines insights: bladder cancer, version 5.2018. J Natl Compr Cancer Netw.

[37] Flaig TW (2020). Bladder cancer, version 3.2020, NCCN clinical practice guidelines in oncology. J Natl Compr Cancer Netw.

[38] Charlton ME (2014). Bladder cancer collaborative stage variables and their data quality, usage, and clinical implications: a review of SEER data, 2004–2010. Cancer.

[39] Matsuo K (2018). Trends of low-grade serous ovarian carcinoma in the United States. J Gynecol Oncol.

[40] Lopez-Beltran A (2019). Variants and new entities of bladder cancer. Histopathology.

[41] Zhang L, Sanagapalli S, Stoita A (2018). Challenges in diagnosis of pancreatic cancer. World J Gastroenterol.

[42] de Leon AD, Pedrosa I (2017). Imaging and screening of kidney cancer. Radiol Clin.

[43] Rossi SH (2018). Epidemiology and screening for renal cancer. World J Urol.

[44] Henrikson NB (2019). Screening for pancreatic cancer: updated evidence report and systematic review for the US Preventive Services Task Force. JAMA.

[45] Canto MI (2013). International Cancer of the Pancreas Screening (CAPS) Consortium summit on the management of patients with increased risk for familial pancreatic cancer. Gut.

[46] American Cancer Society (2016). Cancer facts & figures.

[47] Gray RE, Harris GT (2019). Renal cell carcinoma: diagnosis and management. Am Fam Phys.

[48] IARC Working Group Reports (2014) Helicobacter pylori eradication as a strategy for preventing gastric cancer. IARC working group reports

[49] Areia M (2013). Screening for gastric cancer and surveillance of premalignant lesions: a systematic review of cost-effectiveness studies. Helicobacter.

[50] Saumoy M (2018). Cost effectiveness of gastric cancer screening according to race and ethnicity. Gastroenterology.

[51] He H (2020). Trends in the incidence and survival of patients with esophageal cancer: a SEER database analysis. Thoracic Cancer.

[52] Macías-García F, Domínguez-Muñoz JE (2016). Update on management of Barrett's esophagus. World J Gastrointest Pharmacol Therap.

[53] Arnal MJD, Arenas ÁF, Arbeloa ÁL (2015). Esophageal cancer: risk factors, screening and endoscopic treatment in Western and Eastern countries. World J Gastroenterol.

[54] Vajravelu RK (2021). Characterization of prevalent, post-endoscopy, and incident esophageal cancer in the United States: a large retrospective cohort study. Clin Gastroenterol Hepatol.

[55] Hollingsworth JM (2006). Rising incidence of small renal masses: a need to reassess treatment effect. J Natl Cancer Inst.

[56] Zahnd WE (2018). Rural–urban differences in cancer incidence and trends in the United States. Cancer Epidemiol Biomark Prev.

[57] Henley SJ (2017). Invasive cancer incidence, 2004–2013, and deaths, 2006–2015, in nonmetropolitan and metropolitan counties—United States. MMWR Surveill Summ.

[58] O’Keefe EB, Meltzer JP, Bethea TN (2015). Health disparities and cancer: racial disparities in cancer mortality in the United States, 2000–2010. Front Public Health.

[59] Singh GK, Jemal A (2017). Socioeconomic and racial/ethnic disparities in cancer mortality, incidence, and survival in the United States, 1950–2014: over six decades of changing patterns and widening inequalities. J Environ Public Health.

[60] Phillips SM (2015). Survivors of childhood cancer in the United States: prevalence and burden of morbidity. Cancer Epidemiol Prev Biomark.

[61] Walko CM, McLeod HL (2014). Personalizing medicine in geriatric oncology. J Clin Oncol.

[62] Rowland JH, Bellizzi KM (2014). Cancer survivorship issues: life after treatment and implications for an aging population. J Clin Oncol.

