# Supplementary material for: Underlying mechanisms of change in cancer prevalence in older U.S. adults: contributions of incidence, survival, and ascertainment at early stages
Source: Cancer Causes Control. 2022 Jul 7;33(9):1161–72. doi: 10.1007/s10552-022-01595-6 (PMC9360135; doi:10.1007/s10552-022-01595-6)
Supplement: Supplementary file 1 — (PDF 221 kb) [file 10552_2022_1595_MOESM1_ESM.pdf]

Lung Cancer

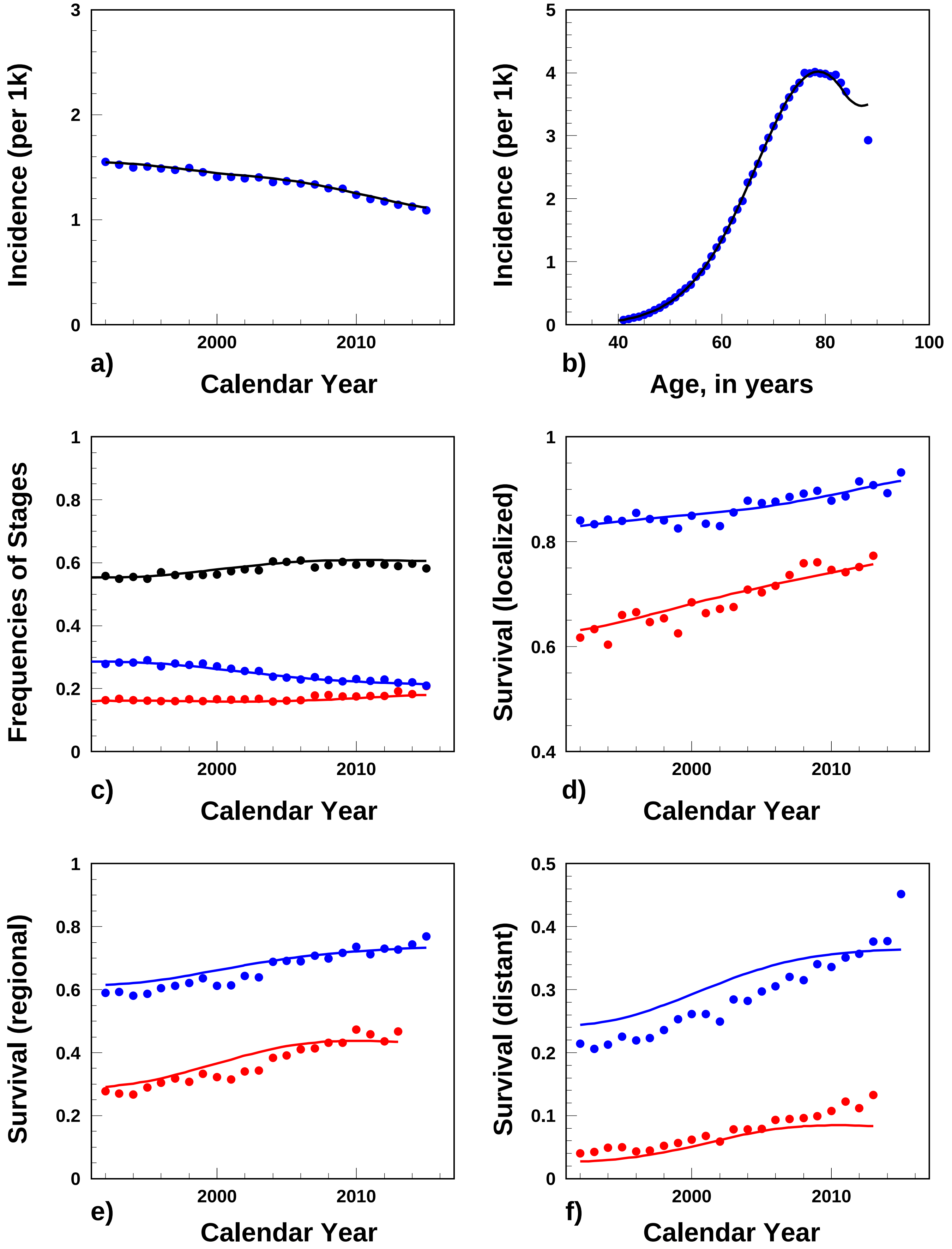
a)
Incidence (per 1k)
Calendar Year
b)
Incidence (per 1k)
Age, in years
c)
Frequencies of Stages
Calendar Year
d)
Survival (localized)
Calendar Year
e)
Survival (regional)
Calendar Year
f)
Survival (distant)
Calendar Year

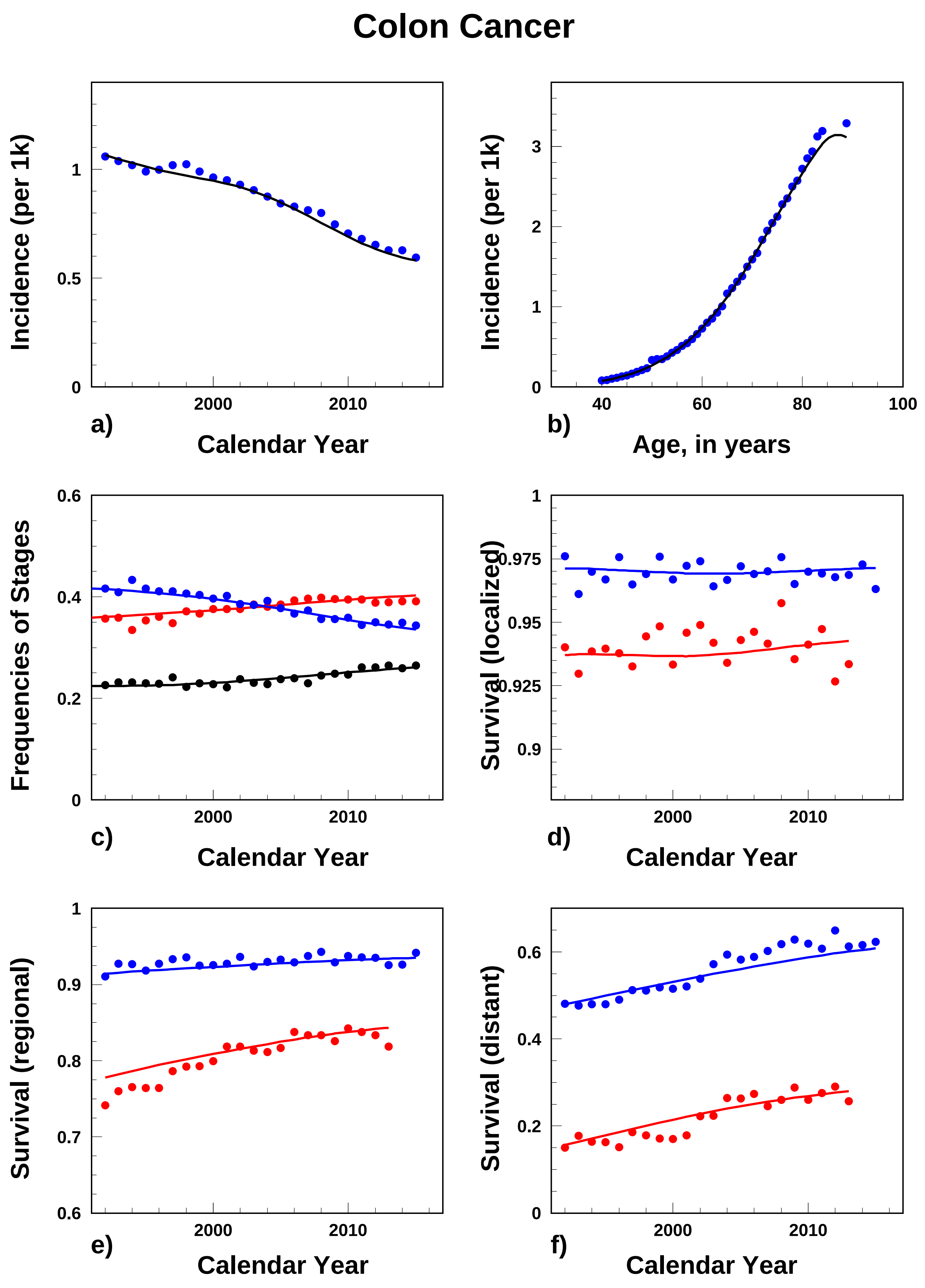
Colon Cancer
Incidence (per 1k)
a)
Calendar Year
Incidence (per 1k)
b)
Age, in years
Frequencies of Stages
c)
Calendar Year
Survival (localized)
d)
Calendar Year
Survival (regional)
e)
Calendar Year
Survival (distant)
f)
Calendar Year

Pancreas Cancer

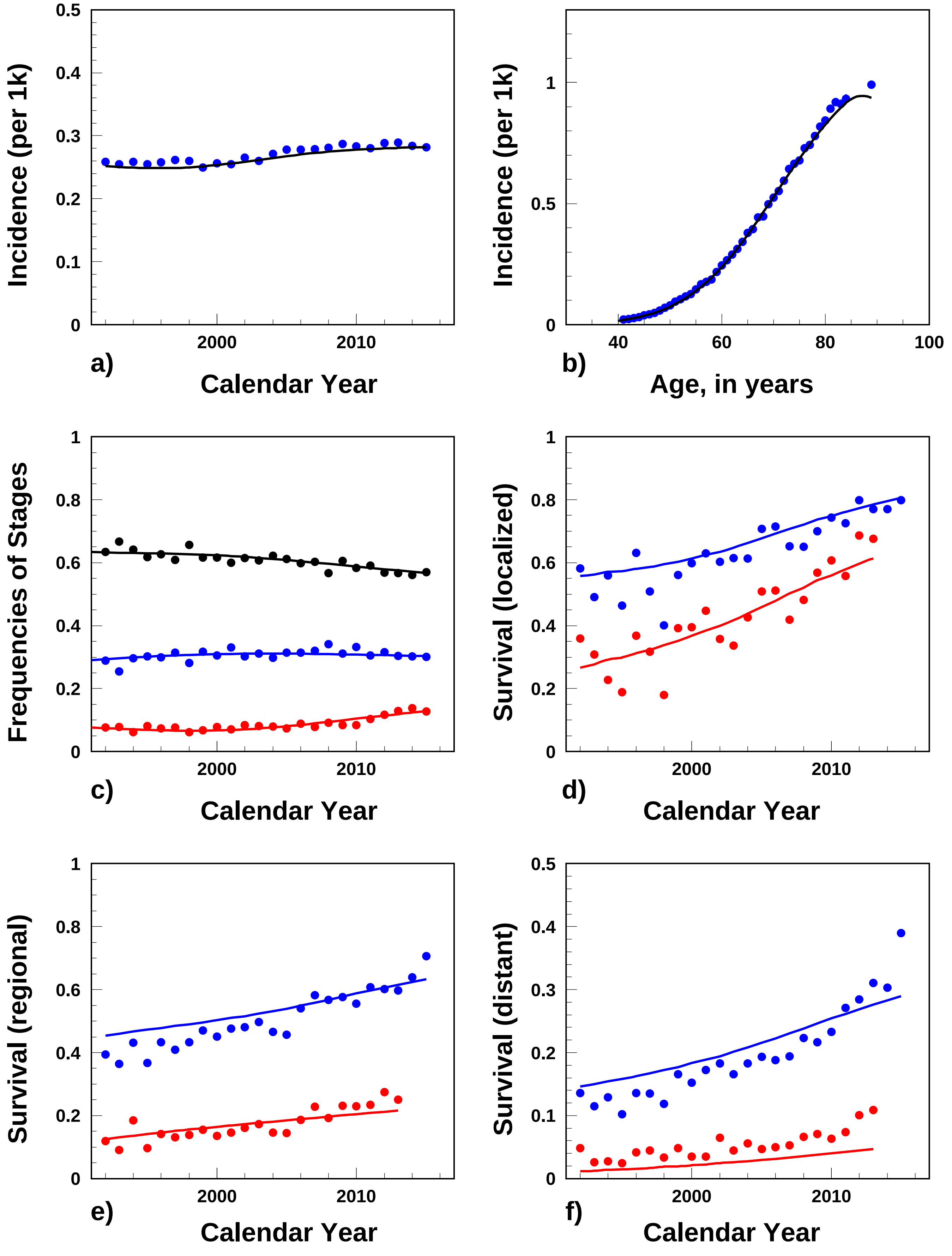

Bladder Cancer

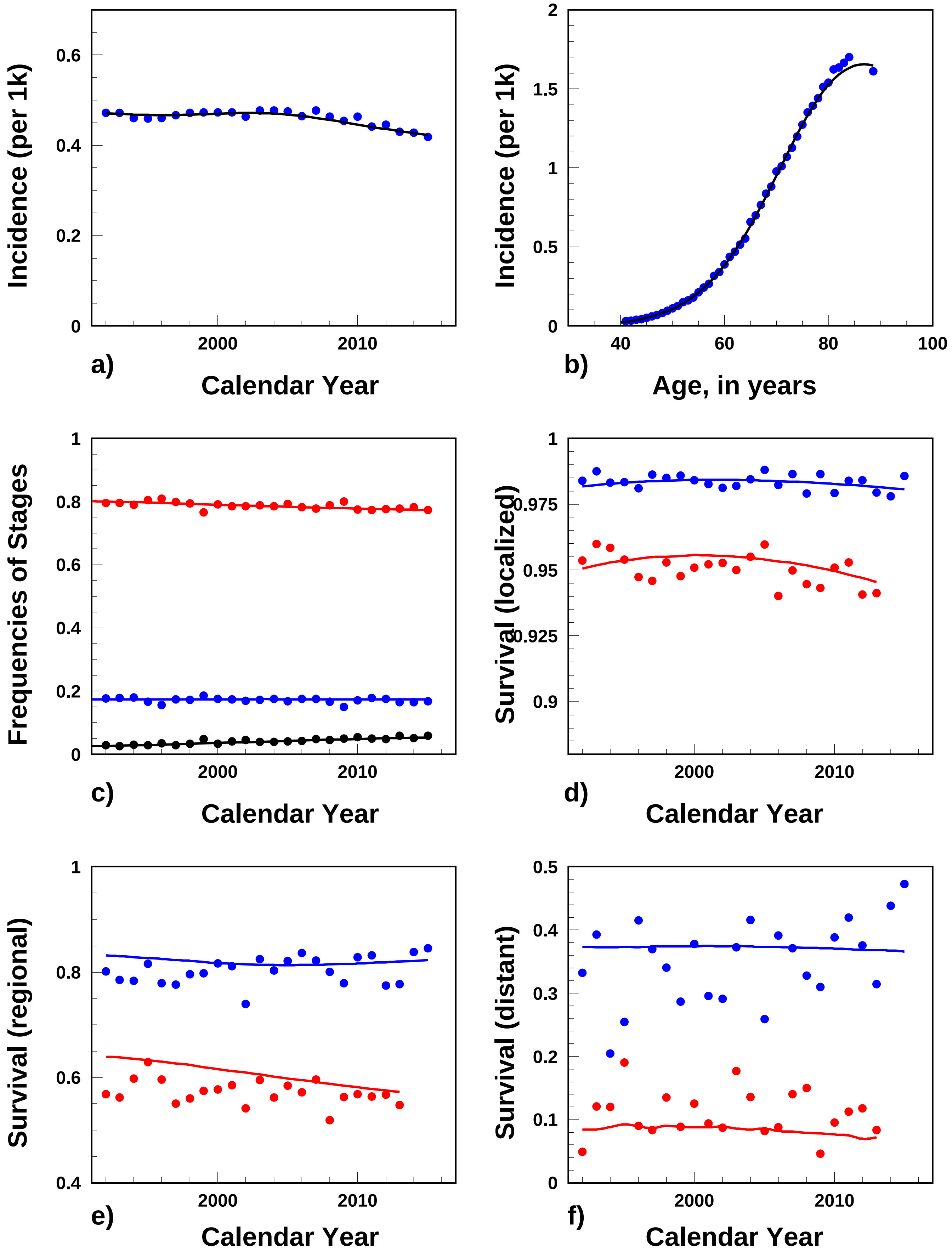

Esophagus Cancer

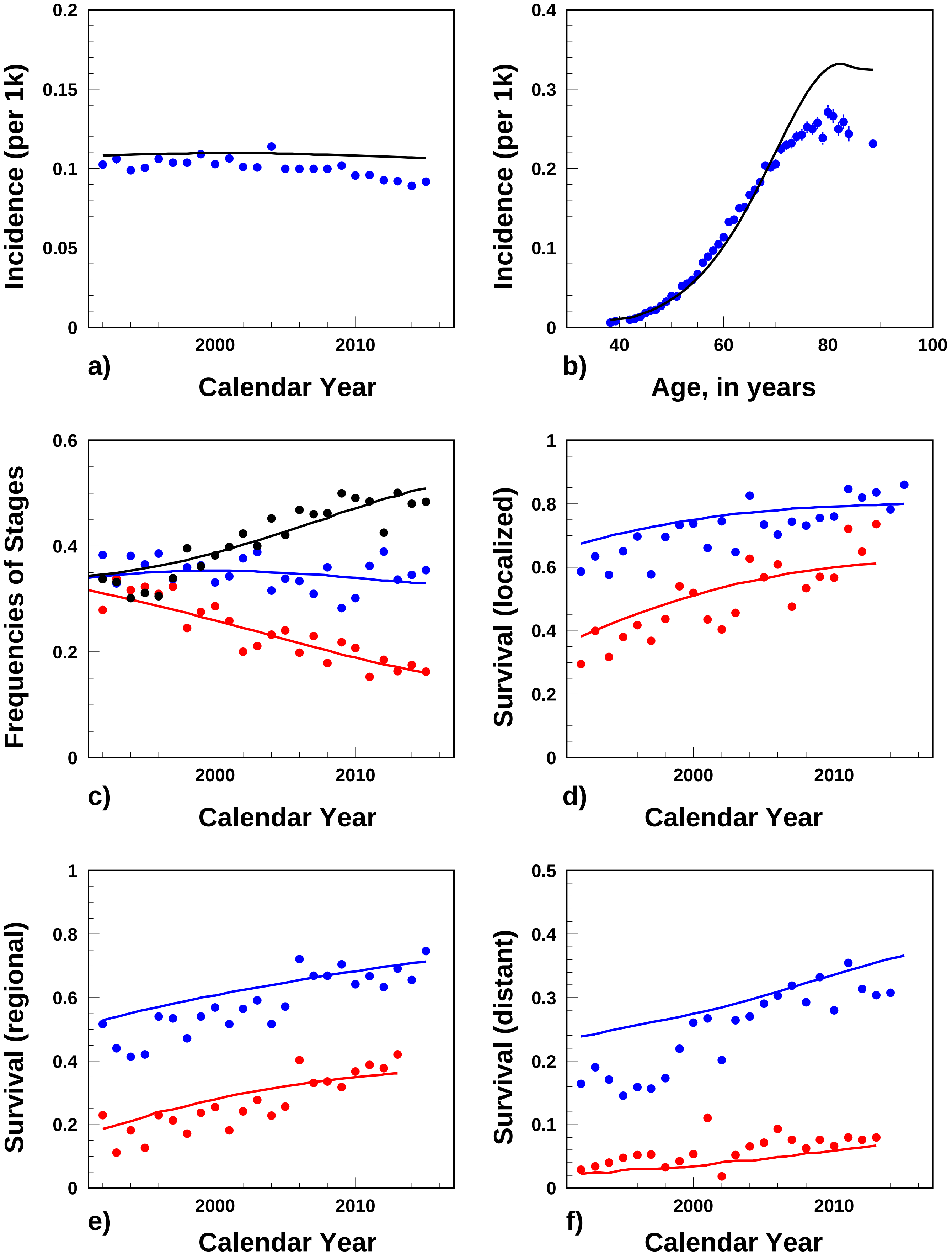

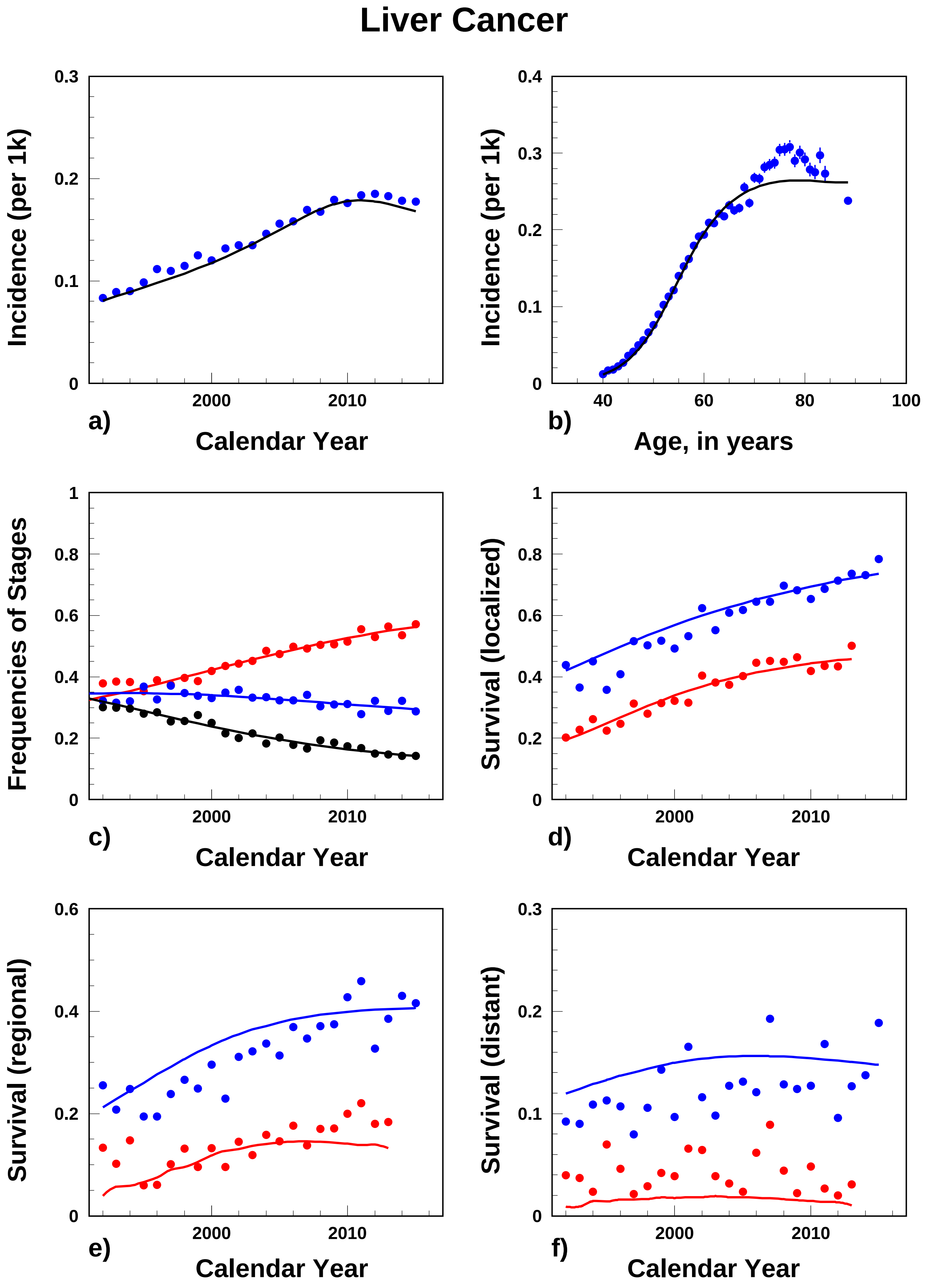
Liver Cancer
Incidence (per 1k)
a)
Calendar Year
Incidence (per 1k)
b)
Age, in years
Frequencies of Stages
c)
Calendar Year
Survival (localized)
d)
Calendar Year
Survival (regional)
e)
Calendar Year
Survival (distant)
f)
Calendar Year

Kidney Cancer

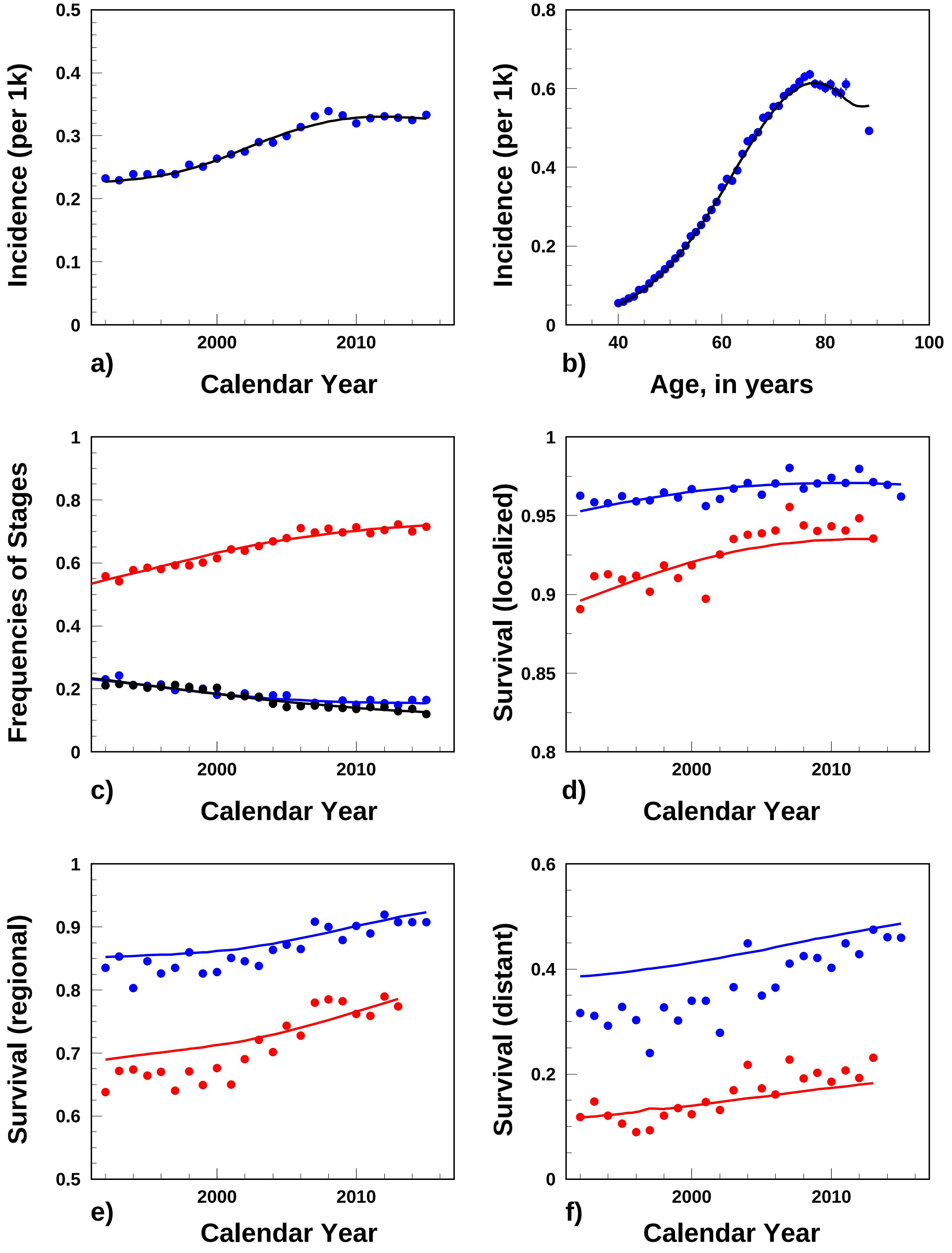

Stomach Cancer

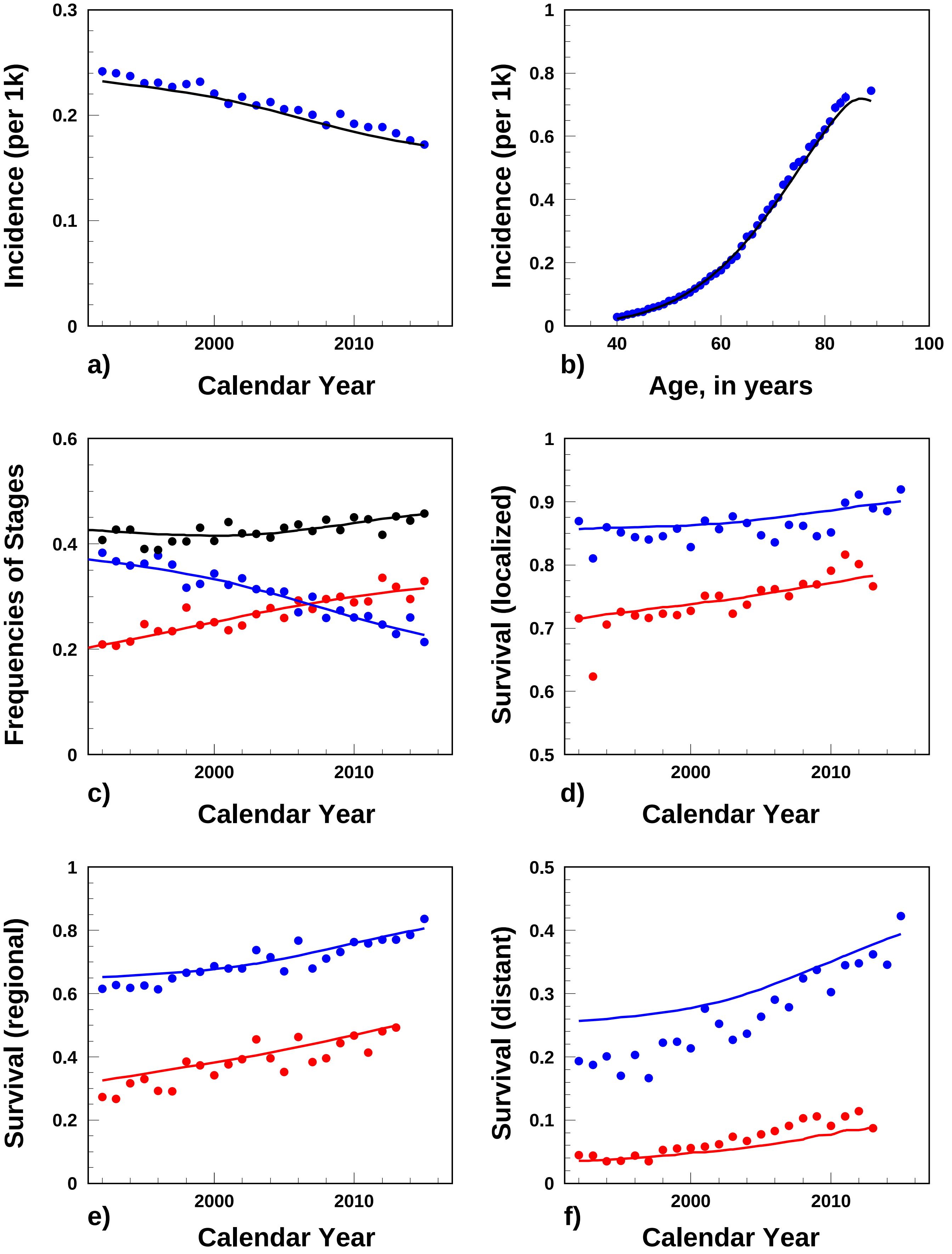

Ovarian Cancer

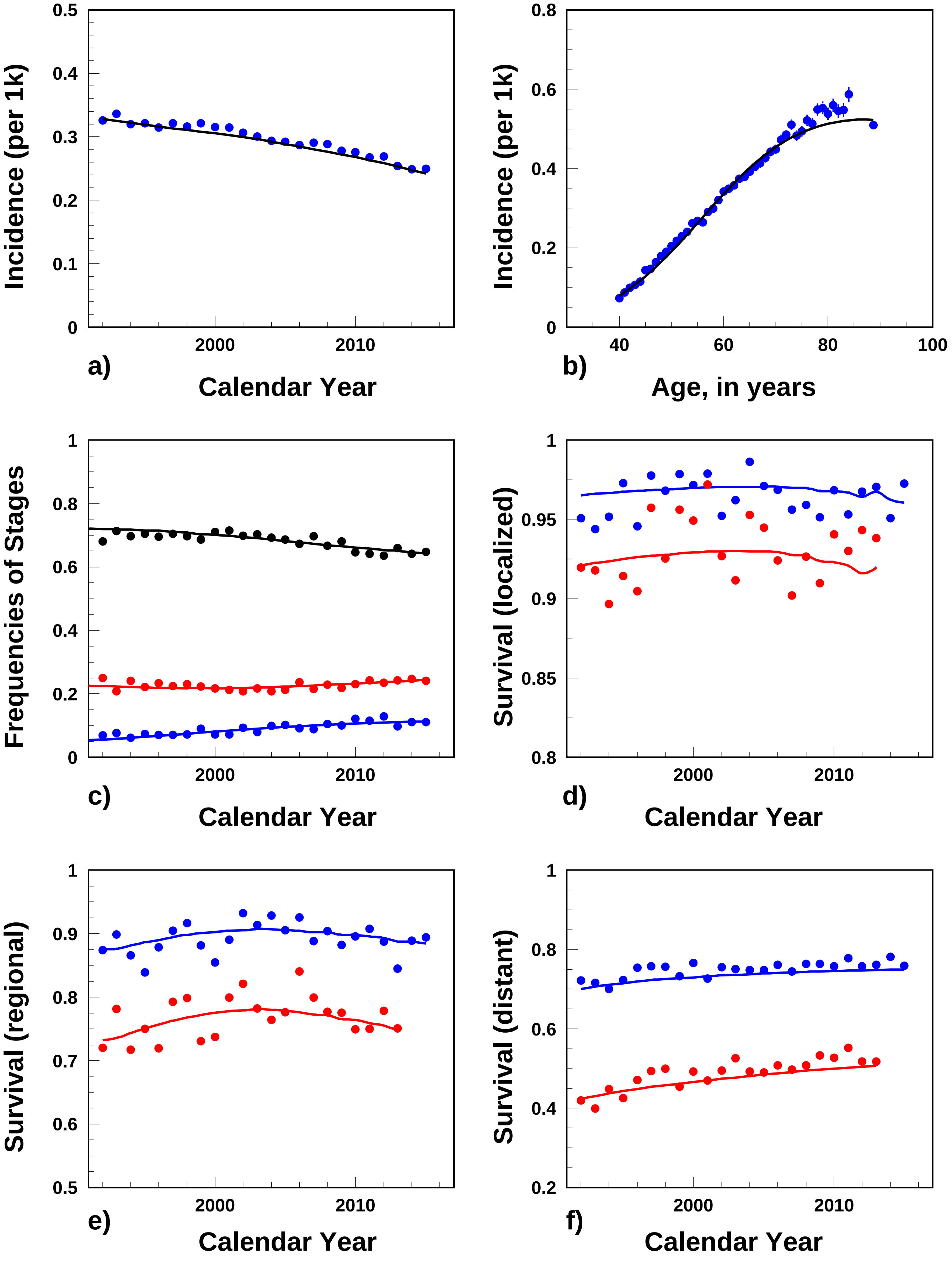

Female Breast Cancer

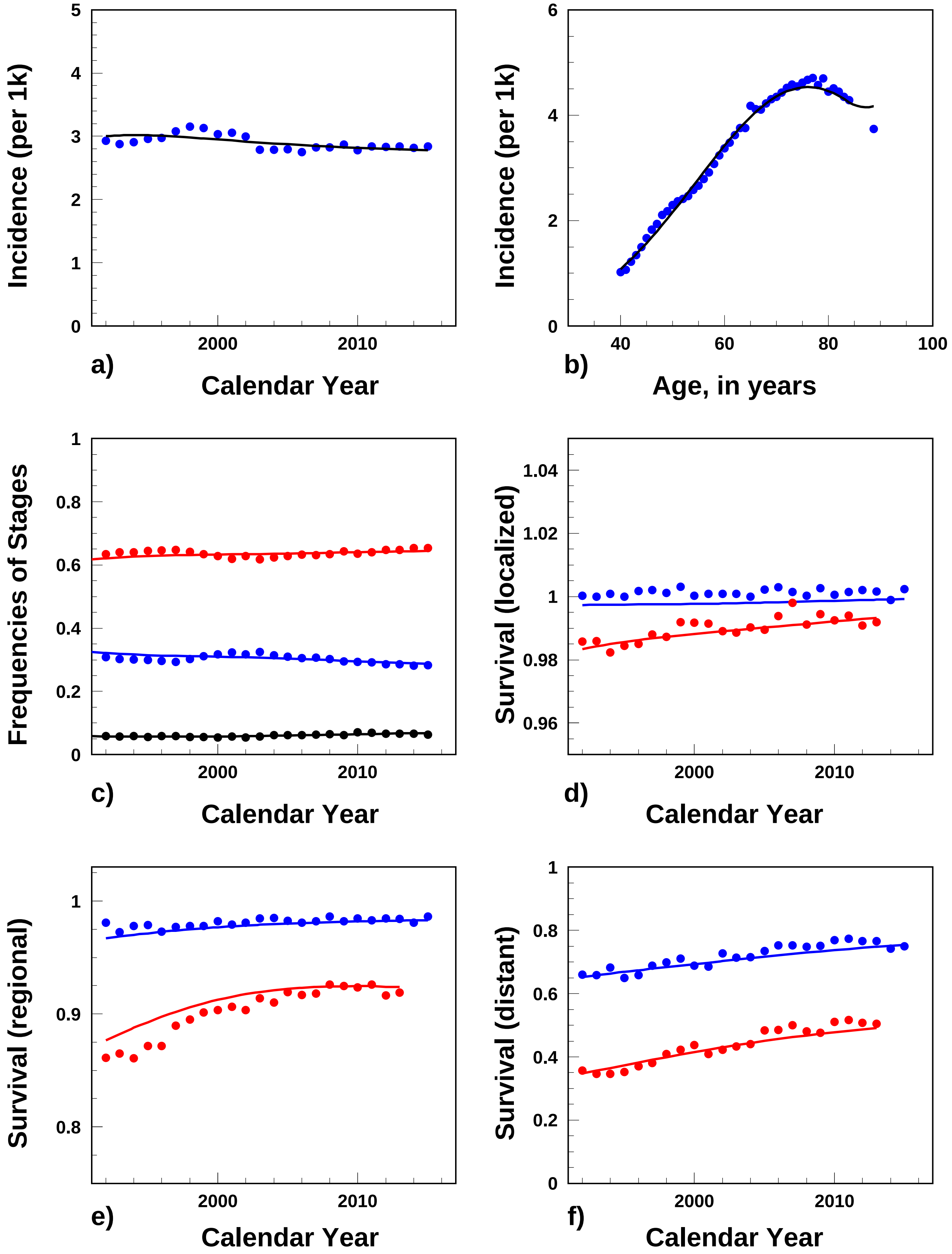

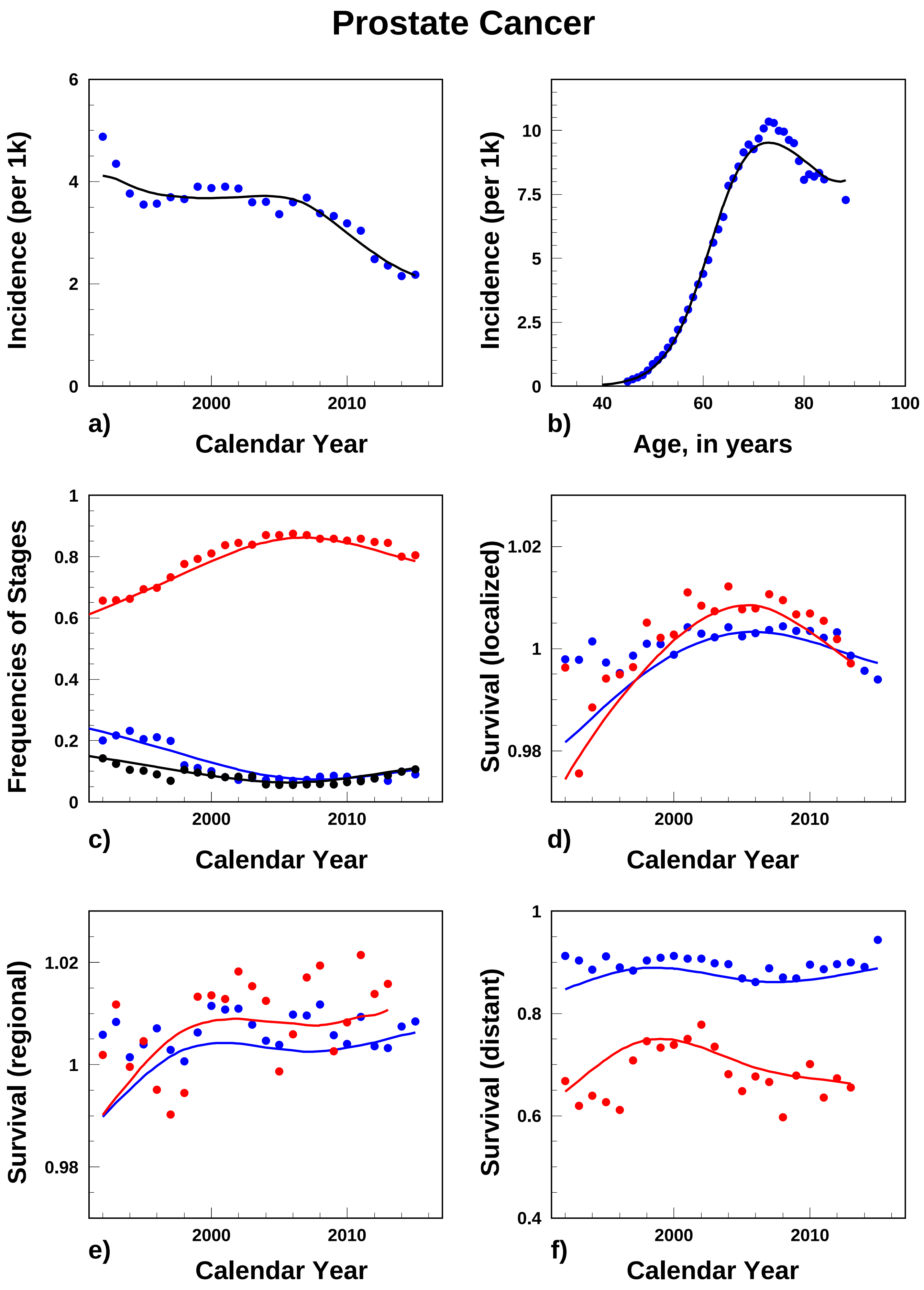
Prostate Cancer
Incidence (per 1k)
a)
Calendar Year
Incidence (per 1k)
b)
Age, in years
Frequencies of Stages
c)
Calendar Year
Survival (localized)
d)
Calendar Year
Survival (regional)
e)
Calendar Year
Survival (distant)
f)
Calendar Year

Melanoma

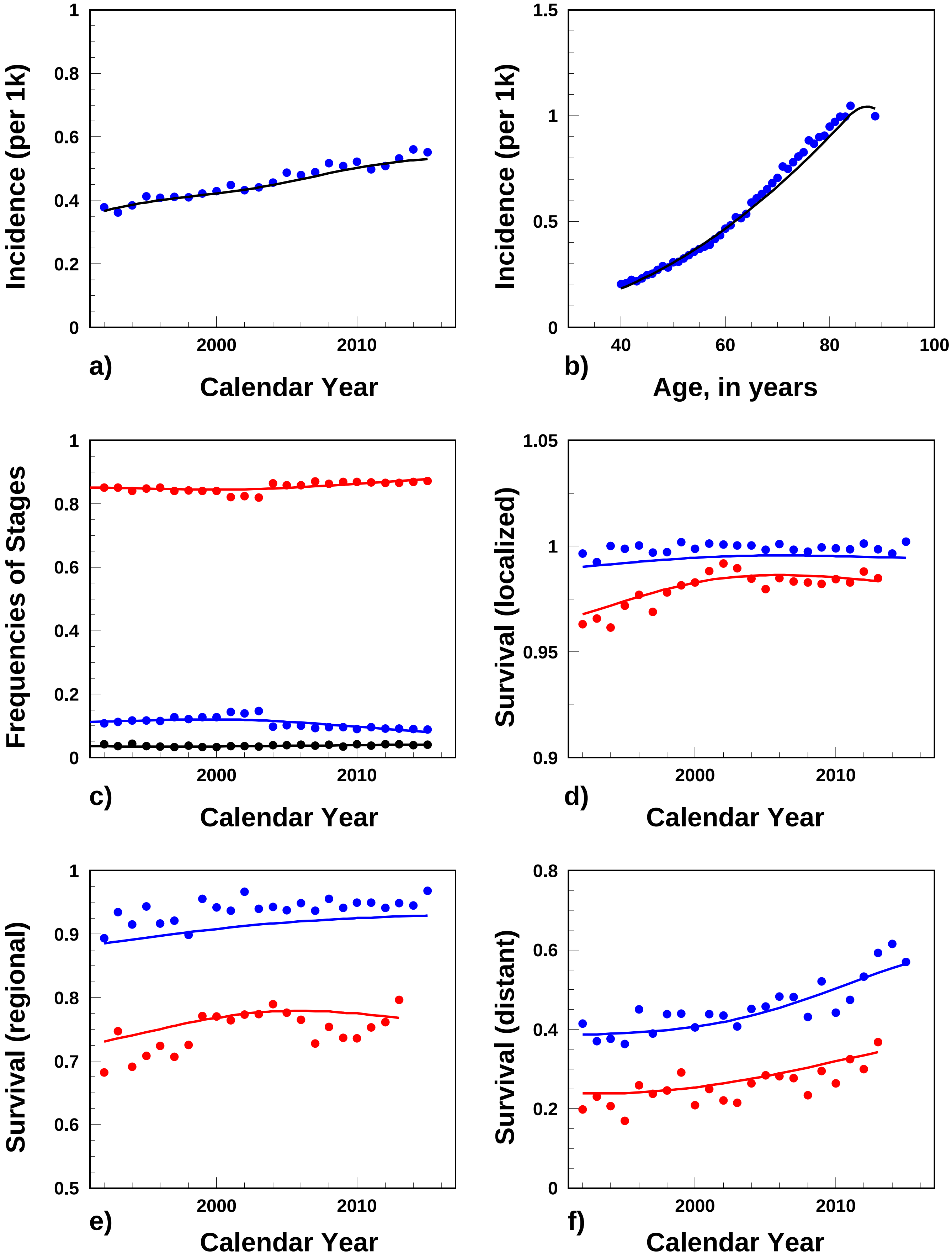

Incidence (per 1k)
0
0.2
0.4
0.6
0.8
1
2000
2010
a)
Calendar Year
Incidence (per 1k)
0
0.5
1
1.5
40
60
80
100
b)
Age, in years
Frequencies of Stages
0
0.2
0.4
0.6
0.8
1
2000
2010
c)
Calendar Year
Survival (localized)
0.9
0.95
1
1.05
2000
2010
d)
Calendar Year
Survival (regional)
0.5
0.6
0.7
0.8
0.9
1
2000
2010
e)
Calendar Year
Survival (distant)
0
0.2
0.4
0.6
0.8
2000
2010
f)
Calendar Year
